# Efficacy and safety of durable versus biodegradable polymer drug-eluting stents in patients with acute myocardial infarction complicated by cardiogenic shock

**DOI:** 10.1038/s41598-024-56925-2

**Published:** 2024-03-15

**Authors:** Woo Jin Jang, Ik Hyun Park, Ju Hyeon Oh, Ki Hong Choi, Young Bin Song, Joo-Yong Hahn, Seung-Hyuk Choi, Hyeon-Cheol Gwon, Chul-Min Ahn, Cheol Woong Yu, Hyun-Joong Kim, Jang-Whan Bae, Sung Uk Kwon, Hyun Jong Lee, Wang Soo Lee, Jin-Ok Jeong, Sang-Don Park, Jeong Hoon Yang

**Affiliations:** 1 Division of Cardiology, Chung-Ang University Hospital, Seoul, Republic of Korea; 2 Department of Cardiology, Samsung Changwon Hospital, Sungkyunkwan University School of Medicine, Changwon, Republic of Korea; 3 Division of Cardiology, Heart Vascular Stroke Institute, Samsung Medical Center, Sungkyunkwan University School of Medicine, Seoul, Republic of Korea; 4 Division of Cardiology, Severance Cardiovascular Hospital, Yonsei University College of Medicine, Seoul, Republic of Korea; 5 Division of Cardiology, Department of Internal Medicine, Korea University Anam Hospital, Seoul, Republic of Korea; 6 Division of Cardiology, Konkuk University Medical Center, Seoul, Republic of Korea; 7 Division of Cardiology, Chungbuk National University College of Medicine, Cheongju, Republic of Korea; 8 Division of Cardiology, Ilsan Paik Hospital, University of Inje College of Medicine, Seoul, Republic of Korea; 9 Division of Cardiology, Sejong General Hospital, Bucheon, Republic of Korea; 10 Division of Cardiology, Chungnam National University Hospital, Daejeon, Republic of Korea; 11 Division of Cardiology, Inha University Hospital, Incheon, Republic of Korea; 12 Division of Cardiology, Department of Critical Care Medicine and Medicine, Samsung Medical Center, Sungkyunkwan University School of Medicine, 81 Irwon-ro, Gangnam-gu, Seoul, 135-710 Republic of Korea

**Keywords:** Polymers, Drug-eluting stent, Cardiogenic shock, Myocardial infarction, Interventional cardiology, Cardiovascular diseases

## Abstract

The clinical impact of different polymer technologies in newer-generation drug-eluting stents (DESs) for patients with acute myocardial infarction (AMI) complicated by cardiogenic shock (CS) remains poorly understood. We investigated the efficacy and safety of durable polymer DESs (DP-DESs) compared with biodegradable polymer DESs (BP-DESs). A total of 620 patients who underwent percutaneous coronary intervention with newer-generation DESs for AMI complicated by CS was divided into two groups based on polymer technology: the DP-DES group (n = 374) and the BP-DES group (n = 246). The primary outcome was target vessel failure (TVF) during a 12-month follow-up, defined as a composite of cardiac death, myocardial infarction, or target vessel revascularization. Both the DP-DES and BP-DES groups exhibited low stent thrombosis rates (1.3% vs. 1.6%, p = 0.660). The risk of TVF did not significantly differ between the two groups (34.2% vs. 28.5%, hazard ratio [HR] 0.94, 95% confidence interval [CI] 0.69–1.29, *p* = 0.721). This finding remained consistent after adjustment with inverse probability of treatment weighting (28.1% vs. 25.1%, HR 0.98, 95% CI 0.77–1.27, *p* = 0.899). In AMI patients complicated by CS, the risk of a composite of cardiac death, myocardial infarction, or target vessel revascularization was not significantly different between those treated with DP-DESs and those treated with BP-DESs.

**Trial registration:** RESCUE registry, https://clinicaltrials.gov/ct2/show/NCT02985008, NCT02985008.

## Introduction

The development of antiproliferative drugs that could be released from a polymer matrix surrounding stent struts has led to concerns about the impact of durable polymers on the performance of drug-eluting stents (DESs)^1^. Chronic inflammation resulting from the durable polymers was found to impair endothelialization on the stent strut, increasing the likelihood of late stent thrombosis^1^. Moreover, the polymers were found to contribute to stent-related adverse events by exacerbating the inflammatory response and delaying arterial healing^2,3^. In response to these issues, biodegradable polymer DESs (BP-DESs) were developed that dissolve over time, leaving only the bare-metal stent behind. These new DESs have been shown to be safer and to result in better patient-oriented outcomes than the first-generation durable polymer DESs (DP-DESs)^4^. Meanwhile, several recent studies have indicated that contemporary DP-DESs using biocompatible polymers are thrombo-resistant and have comparable efficacy and safety to BP-DESs, even in patients with acute coronary syndrome (ACS)^5,6^.

In the setting of acute myocardial infarction (AMI) complicated by cardiogenic shock (CS), a condition characterized by high acuity, potential fatality, and diverse hemodynamics resulting in end-organ hypoperfusion, as well as a significant thrombus burden with the potential for undersized stenting^7,8^, an area of great uncertainty is which coronary stents perform better in such a critical setting. Particularly, in the newer-generation DES era, limited data were available on comparison of the efficacy and safety between DESs and the prognostic role of polymer technology in AMI patients complicated by CS. Thus, we sought to compare the efficacy and safety of DP-DES with BP-DES in AMI patients complicated by CS who underwent percutaneous coronary intervention (PCI) with newer-generation DESs, based on data from a dedicated, large-scale CS registry.

## Methods

### Ethical statement

The study protocol received approval from the Ethics Committee of the Samsung Medical Center with approval number 2016-03-130 on April 6, 2016. Additionally, the study was approved by the local ethics committees of all the participating study centers. The study followed the principles of the Declaration of Helsinki. For retrospectively enrolled patients, the requirement for informed consent was waived by the institutional review boards (IRBs) of the participating hospitals (Seoul Hospital, Ewha Womans University College of Medicine IRB; Samsung Changwon Hospital, Sungkyunkwan University School of Medicine IRB; Samsung Medical Center, Sungkyunkwan University School of Medicine IRB; Severance Cardiovascular Hospital, Yonsei University College of Medicine IRB; Korea University Anam Hospital IRB; Konkuk University Medical Center IRB; Chungbuk National University College of Medicine IRB; Ilsan Paik Hospital, University of Inje College of Medicine IRB; Sejong General Hospital IRB; Chung-Ang University Hospital IRB; Chungnam National University Hospital IRB; Inha University Hospital IRB). All patients enrolled prospectively provided written informed consent prior to enrollment.

### Study population

**Figure 1:**
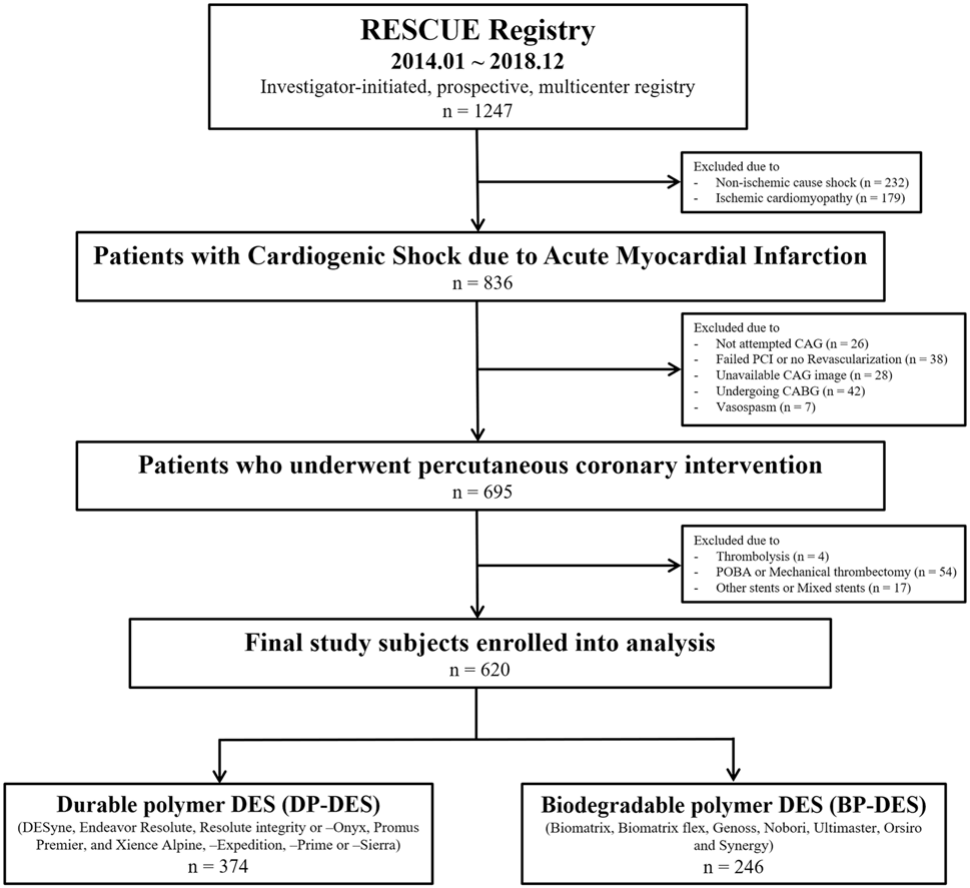
Schematic illustration of study cohort selection. *BMS* bare-metal stent, *CABG* coronary artery bypass grafting, *CAG* coronary angiography, *DES* drug-eluting stent, *PCI* percutaneous coronary intervention, *POBA* percutaneous only balloon angioplasty.

The RESCUE (REtrospective and prospective observational Study to investigate Clinical oUtcomes and Efficacy of left ventricular assist device for Korean patients with cardiogenic shock, NCT02985008) registry was designed according to previous descriptions^9^. From January 2014 to December 2018, a total of 1,247 consecutive patients aged over 19 years with CS was enrolled from 12 tertiary centers in the Republic of Korea. The inclusion criteria for CS were systolic blood pressure measurements of < 90 mmHg for ≥ 30 min or need for vasopressor or inotrope support to maintain a systolic blood pressure > 90 mmHg, along with signs of end-organ hypoperfusion (urine output < 30 mL/h, altered mental status, serum lactate ≥ 2.0 mmol/L, or cold skin) or presence of pulmonary congestion^9^. Major exclusion criteria were refusal of active treatment, out-of-hospital cardiac arrest, and other causes of shock (hypovolemic or septic shock)^9^. Among the 836 AMI patients complicated by CS, 695 who underwent PCI were included in the study after excluding 26 patients who did not undergo coronary angiography, 38 patients for whom revascularization was not performed or PCI for the culprit lesion failed, 28 patients without available coronary angiography images, 42 patients treated with coronary artery bypass grafting, and seven patients with vasospasm. An additional 75 patients were excluded as they were treated with only balloon angioplasty, a bare-metal stent, a stent type used in a small number of patients, or mixed stents. Finally, 620 patients were classified into two groups based on the polymer technology of implanted DESs: the DP-DES group (which included the DESyne [Elixir Medical, Sunnyvale, CA, USA], Endeavor Resolute and Resolute Integrity or -Onyx [Medtronic Vascular, Santa Rosa, CA, USA], Promus Premier [Boston Scientific, Natick, MA, USA], and Xience Alpine, -Expedition, -Prime or -Sierra [Abbott Vascular, Santa Clara, CA, USA] stents; n = 374) and the BP-DES group (which included the Biomatrix and Biomatrix Flex [Biosensors Inc., Newport Beach, CA, USA], Genoss [Genoss Company Limited, Suwon, Korea], Nobori and Ultimaster [Terumo, Tokyo, Japan], Orsiro [Biotronik, Buelach, Switzerland], and Synergy [Boston Scientific, Marlborough, MA, USA] stents; n = 246) (Fig. 1).

### Data collection

The RESCUE registry collected patient demographic information, in-hospital management, procedural and laboratory data, and outcomes using web-based case report forms completed by independent clinical research coordinators. If necessary, further information was retrieved either from medical records or through telephone communication^9^.

### PCI and pharmacologic therapy

PCI was conducted using standard techniques^10^. Anticoagulation during the procedure involved the use of unfractionated heparin or low molecular-weight heparin. The operator had discretion in making decisions regarding predilation or postdilation, thrombus aspiration, and the use of glycoprotein IIb/IIIa inhibitors. There were no limitations on the diameter and length of the stents used. The decision to perform intravascular imaging or fractional flow reserve was at the operator’s discretion. A loading dose of aspirin (300 mg) and P2Y12 inhibitor (clopidogrel 300–600 mg, prasugrel 60 mg, or ticagrelor 180 mg) were administered to all patients, unless they had already received these antiplatelet medications. After the procedure, patients were prescribed indefinite oral aspirin (100 mg once daily) along with either clopidogrel (75 mg once daily), prasugrel (10 mg once daily), or ticagrelor (90 mg twice daily). Additionally, it was recommended that all patients adhere to optimal pharmacological therapy, such as renin-angiotensin system blockade, statins, or beta-blockers, as indicated. The responsible clinicians had the discretion to determine the duration of dual antiplatelet therapy^11,12^.

### Study outcomes and definitions

The definition of clinical events was based on recommendations from the Academic Research Consortium (ARC)^13^. The primary outcome was target vessel failure (TVF), defined as a composite of cardiac death, myocardial infarction, or target vessel revascularization. Secondary outcomes included the individual components comprising the primary outcome, along with all-cause death, any revascularization, re-hospitalization due to heart failure, and definite or probable stent thrombosis. Any unexplained death within the first 30 days after intracoronary stenting was classified as probable stent thrombosis according to the ARC recommendations^13^. Analyses were limited to a 12-month period due to varying durations of follow-up for the DES types.

### Statistical analysis

Categorical variables were reported as count and percentage, and their comparison was performed using Fisher’s exact test or the Chi-square test as suitable. Analysis of continuous variables was performed using Student's t-test or Wilcoxon rank-sum test. For continuous variables that exhibited a normal distribution, Student’s t-test was applied, and the results were presented as mean ± standard deviation. For continuous variables that did not exhibit a normal distribution, Wilcoxon rank-sum test was applied, and the results were presented as median (25–75th percentile). Cumulative event rates were estimated using the Kaplan–Meier method and compared using the log-rank test. Cox proportional hazard models were utilized to calculate the hazard ratio (HR) and 95% confidence interval (CI). The proportional hazards assumption was inspected visually through the “log minus log” plot and was evaluated using Schoenfeld residuals. To address the potential for treatment selection bias based on DES type and other confounding factors, inverse probability of treatment weighting (IPTW) adjustment was performed. The IPTW method was applied using generalized boosted models to estimate treatment effects, and weights were assigned according to the DES type to assess any potential interactions between the two treatment strategies that could have influenced clinical outcomes. To compare the outcomes of the matched groups, we employed stratified and IPTW-adjusted Cox proportional hazard models. All probability values were two-tailed, and *p* < 0.05 was considered statistically significant. Statistical analyses were performed using R version 3.6.0 (R Project for Statistical Computing, Vienna, Austria).

## Results

### Baseline clinical characteristics

Among the 620 patients enrolled in this study, 374 were treated with DP-DES (60.3%, DP-DES group) and 246 with BP-DES (39.6%, BP-DES group) (Fig. 1). Baseline clinical characteristics were not significantly different between the DP-DES group and the BP-DES group except history of previous myocardial infarction (*p* = 0.027), although patients who received DP-DESs had high risk with higher vasoactive inotropic score (*p* = 0.004), serum lactate level (*p* = 0.011), and incidence of mechanical ventilation (*p* < 0.001) or mechanical circulatory support (MCS) (*p* = 0.002) (Table 1). Angiographic and procedural characteristics are shown in Table 2. There were no significant differences in angiographic characteristics such as culprit lesion location, number of diseased vessels or lesions, pre- or post-PCI thrombolysis in myocardial infarction (TIMI) flow of the culprit lesion, or SYNTAX score before or after PCI. In the procedural characteristics, thrombus aspiration was performed more frequently in patients treated with DP-DES than in patients treated with BP-DES (*p* = 0.008).

**Table 1:** Baseline clinical characteristics and In-hospital management.

	Overall population			IPTW population		
	DP-DES	BP-DES	*p* value	DP-DES	BP-DES	*p* value
	n = 374	n = 246		n = 498	n = 491	
Age (years)	67.2 ± 12.4	66.6 ± 12.5	0.550	66.3 ± 12.7	66.3 ± 12.2	0.984
Male	275 (73.5)	178 (72.4)	0.748	363 (72.8)	369 (75.1)	0.325
BMI *(Kg/m*^*2*^*)*	23.7 ± 3.3	24.0 ± 3.6	0.286	23.7 ± 3.2	23.9 ± 3.5	0.544
Clinical presentation			0.062			0.681
NSTEMI	122 (32.6)	63 (25.6)		145 (29.2)	136 (27.7)	
STEMI	252 (67.4)	183 (74.4)		353 (70.8)	355 (72.3)	
Cardiovascular risk factor						
* Hypertension*	204 (54.6)	145 (58.9)	0.280	274 (55.0)	267 (54.3)	0.825
Diabetes mellitus	127 (34.0)	100 (40.7)	0.091	187 (37.6)	182 (37.0)	0.612
Chronic kidney disease	38 (10.2)	16 (6.5)	0.114	42 (8.4)	37 (7.6)	0.742
Current smoker	137 (36.6)	79 (32.1)	0.248	183 (36.8)	160 (32.6)	0.189
Previous PCI	42 (11.2)	28 (11.4)	0.953	52 (10.5)	51 (10.3)	0.917
Previous myocardial infarction	56 (15.0)	22 (8.9)	0.027	65 (13.0)	51 (10.4)	0.472
Peripheral artery disease	11 (2.9)	7 (2.9)	0.945	14 (2.8)	16 (3.2)	0.900
Previous history of stroke	28 (7.5)	22 (8.9)	0.515	39 (7.9)	39 (8.0)	0.493
Left ventricular ejection fraction *(%)*	37.3 ± 15.0	37.1 ± 15.6	0.890	37.0 ± 14.9	36.9 ± 15.8	0.867
Systolic blood pressure, *mmHg*	73.1 ± 28.8	77.3 ± 30.2	0.079	76.6 ± 28.2	75.4 ± 30.6	0.546
Diastolic blood pressure, *mmHg*	46.9 ± 20.0	49.0 ± 19.3	0.195	49.1 ± 19.9	48.0 ± 19.7	0.351
Heart rate, beat/min	79.0 ± 34.1	77.1 ± 31.1	0.484	79.8 ± 33.4	76.2 ± 33.4	0.095
Laboratory findings						
Hemoglobin, *g/dL*	13.1 ± 2.4	13.1 ± 2.2	0.960	13.2 ± 2.3	13.2 ± 2.1	0.819
Creatinine, *mg/dL*	1.4 ± 1.1	1.6 ± 1.6	0.265	1.4 ± 1.1	1.4 ± 1.2	0.972
Glucose, *mg/dL*	230.8 ± 119.2	236.3 ± 123.0	0.589	237.0 ± 134.6	237.9 ± 115.0	0.914
Lactate, *mmol/L*	1.8 (1.3 3.8)	1.5 (0.9–2.8)	0.011	1.7 (1.1–3.2)	1.5 (0.9–2.7)	0.385
Peak CK-MB, ng/mL	180.1 (50.0–300.0)	205.9 (62.8–300.0)	0.641	200.0 (63.2–300.0)	221.6 (52.4–300.0)	0.504
Peak Troponin I, *ng/mL*	18.4 (2.0–58.2)	14.2 (1.1–66.3)	0.283	19.2 (2.5–60.0)	16.6 (1.5–78.2)	0.433
Undergoing CPR	81 (21.7)	39 (15.9)	0.074	89 (17.8)	96 (19.6)	0.355
Vasoactive Inotropic Score	30.5 (10.0–83.6)	20.0 (7.1–80.0)	0.004	30.0 (10.0–75.0)	26.6 (9.5–97.0)	0.805
In-hospital management						
Mechanical ventilation	229 (61.2)	108 (43.9)	< 0.001	265 (53.3)	261 (53.1)	0.494
Renal-replacement therapy	71 (19.0)	35 (14.2)	0.124	80 (16.0)	78 (15.9)	0.759
Mechanical circulatory support	234 (62.6)	123 (50.0)	0.002	286 (57.4)	276 (56.2)	0.876

Data are n (%), mean ± standard deviation, or median (interquartile range).

*BP-DES* biodegradable polymer drug-eluting stent, *BMI* body-mass index, *CABG* coronary artery bypass grafting, *CK-MB* creatine kinase myocardial band, *CPR* cardiopulmonary resuscitation, *DP-DES* durable polymer drug-eluting stent, *IPTW* inverse probability of treatment weighting, *PCI* percutaneous coronary intervention, *STEMI* ST-segment elevation myocardial infarction, *NSTEMI* nonST-segment elevation myocardial infarction.

**Table 2:** Angiographic and Procedural characteristics.

	Overall population			IPTW population		
	DP-DES	BP-DES	*p* value	DP-DES	BP-DES	*p* value
	n = 374	n = 246		n = 498	n = 491	
Angiographic findings						
Culprit lesion location			0.074			0.109
LM	63 (16.8)	31 (12.6)		74 (14.9)	71 (14.5)	
LAD	160 (42.8)	102 (41.5)		216 (43.2)	194 (39.4)	
LCX	41 (11.0)	19 (7.7)		54 (10.8)	41 (8.3)	
RCA	110 (29.4)	94 (38.2)		155 (31.1)	185 (37.7)	
Culprit lesion TIMI flow grade, pre-PCI			0.694			0.987
0	209 (55.9)	147 (59.8)		289 (58.0)	287 (58.4)	
1	33 (8.8)	17 (6.9)		41 (8.2)	42 (8.6)	
2	60 (16.0)	40 (16.3)		75 (15.0)	73 (14.8)	
3	72 (19.3)	42 (17.1)		94 (18.9)	89 (18.2)	
Culprit lesion TIMI flow grade, post-PCI			0.120			0.975
0	4 (1.1)	3 (1.2)		4 (0.9)	3 (0.7)	
1	4 (1.1)	9 (3.7)		9 (1.8)	10 (2.1)	
2	46 (12.3)	36 (14.6)		53 (10.6)	52 (10.5)	
3	320 (85.6)	198 (80.5)		432 (86.7)	426 (86.7)	
Vessel disease			0.483			0.183
1-vessel disease	76 (20.3)	57 (23.2)		110 (22.0)	105 (21.5)	
2-vessel disease	168 (44.9)	99 (40.2)		226 (45.3)	199 (40.5)	
3-vessel disease	130 (34.8)	90 (36.6)		163 (32.7)	187 (38.1)	
Multivessel disease	298 (79.7)	189 (76.8)	0.398	389 (78.0)	386 (78.6)	0.748
SYNTAX score, pre-PCI	23.6 ± 11.0	22.3 ± 10.8	0.140	23.3 ± 10.8	23.2 ± 11.4	0.943
SYNTAX score, post-PCI	6.4 ± 8.2	6.2 ± 7.9	0.672	6.3 ± 7.8	6.5 ± 8.0	0.610
Number of lesions	2.3 ± 1.1	2.3 ± 1.2	0.849	2.3 ± 1.1	2.3 ± 1.1	0.769
Non-culprit LM or proximal LAD involvement	58 (15.5)	39 (15.9)	0.908	85 (17.1)	78 (16.0)	0.957
Procedural characteristics						
Access site			0.222			0.497
Transradial approach	311 (83.2)	195 (79.3)		414 (83.1)	400 (81.4)	
Transfemoral approach	63 (16.8)	51 (20.7)		84 (16.9)	91 (18.6)	
Contrast volume, mL	177.8 ± 88.5	184.8 ± 61.5	0.567	185.8 ± 100.8	186.0 ± 59.5	0.990
Thrombus aspiration	102 (27.3)	92 (37.4)	0.008	149 (29.9)	148 (30.2)	0.728
Performed staged PCI	38 (10.2)	23 (9.4)	0.932	48 (9.7)	52 (10.6)	0.790
Timing of staged PCI, *days*	4.0 (3.0–8.0)	4.0 (2.0–9.0)	0.696	4.0 (3.0–7.0)	4.0 (2.0–9.0)	0.730
Number of used stent	1.6 ± 0.9	1.5 ± 0.8	0.140	1.5 ± 0.8	1.5 ± 0.8	0.608
Number of treated lesion	1.5 ± 0.8	1.4 ± 0.7	0.104	1.5 ± 0.8	1.5 ± 0.7	0.433
Maximal diameter of stents, *mm*	3.2 ± 0.5	3.2 ± 0.4	0.561	3.2 ± 0.5	3.2 ± 0.4	0.174
Length of stents, *mm*	38.6 ± 24.4	37.2 ± 24.0	0.484	37.5 ± 23.1	37.5 ± 23.9	0.992

Data are n (%), mean ± standard deviation, or median (interquartile range).

*BP-DES* biodegradable polymer drug-eluting stent, *DP-DES* durable polymer drug-eluting stent, *IPTW* inverse probability of treatment weighting, *LAD* left anterior descending artery, *LCX* left circumflex artery, *LM* left main coronary artery, *PCI* percutaneous coronary intervention, *RCA* right coronary artery, *SYNTAX* Synergy between PCI with Taxus and Cardiac Surgery, *TIMI* thrombolysis in myocardial infarction.

### Clinical outcomes

**Figure 2:**
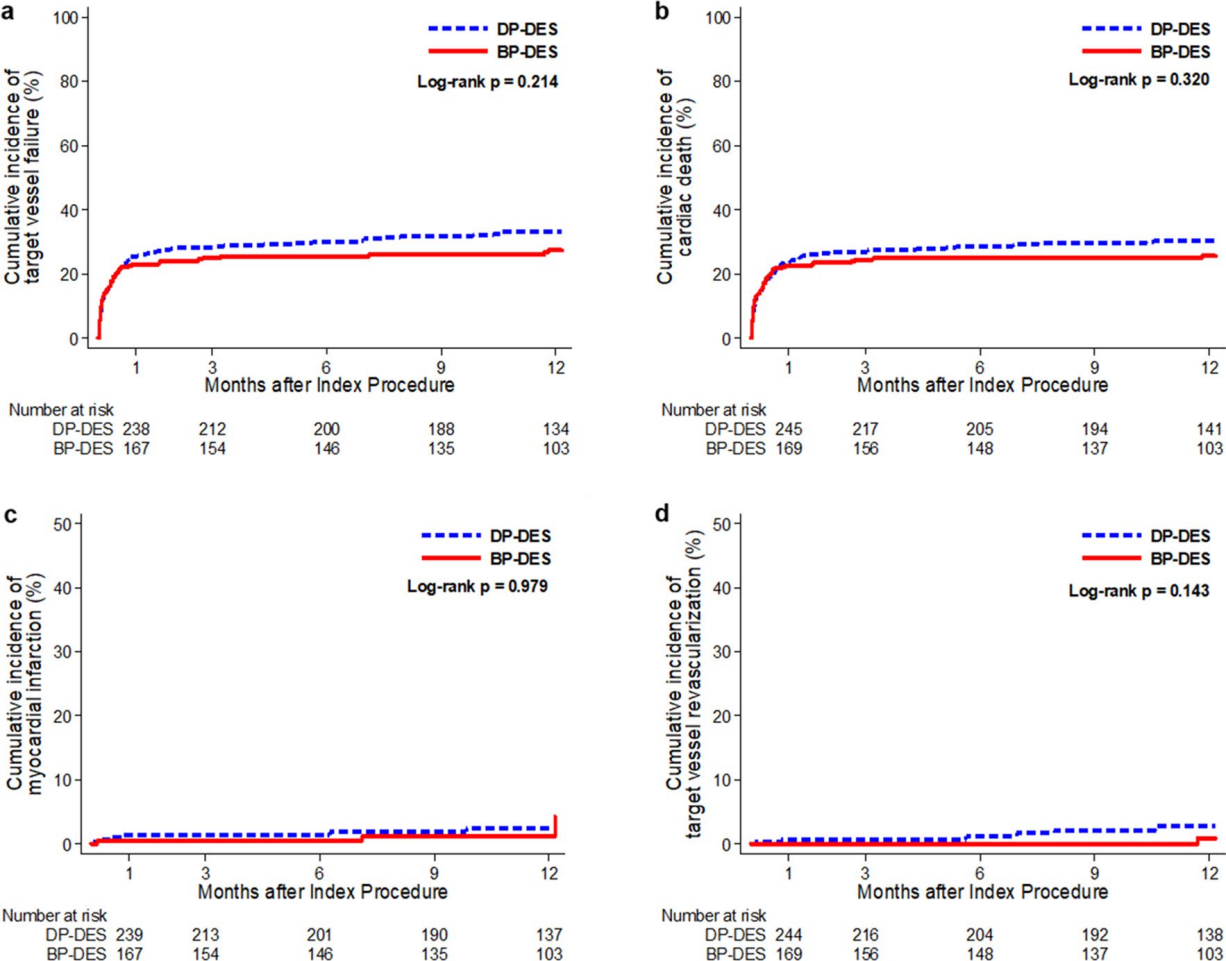
Time-to-event Kaplan–Meier survival curves of primary outcome according to polymer. (**a**) Kaplan–Meier curves for target vessel failure. (**b**) Kaplan–Meier curves for cardiac death. (**c**) Kaplan–Meier curves for myocardial infarction. (**d**) Kaplan–Meier curves for target vessel revascularization. TVF was defined as a composite of cardiac death, MI, and target vessel revascularization. *BP-DES* bioabsorbable polymer drug-eluting stent; *DP-DES* durable polymer drug-eluting stent.

The primary outcome occurred in 128 (34.2%) patients in the DP-DES group and 70 (28.5%) patients in the BP-DES group at 12 months after the index procedure (HR for DP-DES vs. BP-DES 0.94, 95% CI 0.69–1.29; *p* = 0.721) (Table 3 and Fig. 2a). The rate of stent thrombosis was low (1.5%), with most events observed early after the index procedure (a median of 3.0 days). Stent thrombosis occurred in five (1.3%) patients in the DP-DES group and four (1.6%) patients in the BP-DES group (*p* = 0.660) (Table 3). There were no significant differences observed among the individual components comprising the primary outcome (cardiac death 34.0% in the DP-DES group vs. 29.3% in the BP-DES group, *p* = 0.190, Fig. 2b; myocardial infarction 2.4% vs. 2.4%, *p* = 0.999, Fig. 2c; target vessel revascularization 1.6% vs. 1.2%, *p* = 0.602, Fig. 2d), all-cause death (42.0% vs. 33.7%, *p* = 0.103), any revascularization (3.2% vs. 3.7%, *p* = 0.776) and re-hospitalization due to heart failure (5.6% vs. 5.3%, *p* = 0.711) at 12 months (Table 3).

**Table 3:** 12-month Follow-Up Clinical Outcomes According to used stent.

	Overall population				IPTW population			
	DP-DES	BP-DES	Unadjusted HR	*p* value	DP-DES	BP-DES	adjusted HR	*p* value
	n = 374	n = 246	95% CI		n = 498	n = 491	95% CI	
*Target vessel failure	138 (36.9)	78 (31.7)	0.94 (0.69–1.29)	0.721	154 (30.9)	140 (28.6)	0.98 (0.77–1.27)	0.899
All-cause death	157 (42.0)	83 (33.7)	1.26 (0.95–1.67)	0.103	184 (36.7)	157 (32.0)	1.12 (0.90–1.40)	0.296
Cardiac death	127 (34.0)	72 (29.3)	0.81 (0.60–1.11)	0.190	142 (28.5)	127 (25.9)	0.82 (0.64–1.05)	0.121
Myocardial infarction	9 (2.4)	6 (2.4)	0.99 (0.32–3.15)	0.999	6 (1.2)	9 (1.8)	0.91 (0.29–2.89)	0.873
†Stent thrombosis	5 (1.3)	4 (1.6)	0.70 (0.14–3.46)	0.660	6 (1.2)	7 (1.4)	0.81 (0.14–2.83)	0.441
Target vessel revascularization	6 (1.6)	3 (1.2)	1.45 (0.36–5.78)	0.602	7 (1.4)	9 (1.8)	0.87 (0.32–2.32)	0.782
Any revascularization	12 (3.2)	9 (3.7)	0.88 (0.36–2.12)	0.776	16 (3.2)	29 (5.9)	0.55 (0.30–1.03)	0.061
Re-hospitalization due to HF	21 (5.6)	13 (5.3)	1.14 (0.57–2.28)	0.711	26 (5.2)	22 (4.5)	1.24 (0.71–2.17)	0.457

Data are n (%).

*Target vessel failure was defined as a composite of cardiac death, myocardial infarction, and target vessel revascularization.

^†^Definite or probable stent thrombosis.

*BP-DES* biodegradable polymer drug-eluting stent, *CI* confidence interval, *DP-DES* durable polymer drug-eluting stent, *HF* heart failure, *HR* hazard ratio, *IPTW* inverse probability of treatment weighting.

**Figure 3:**
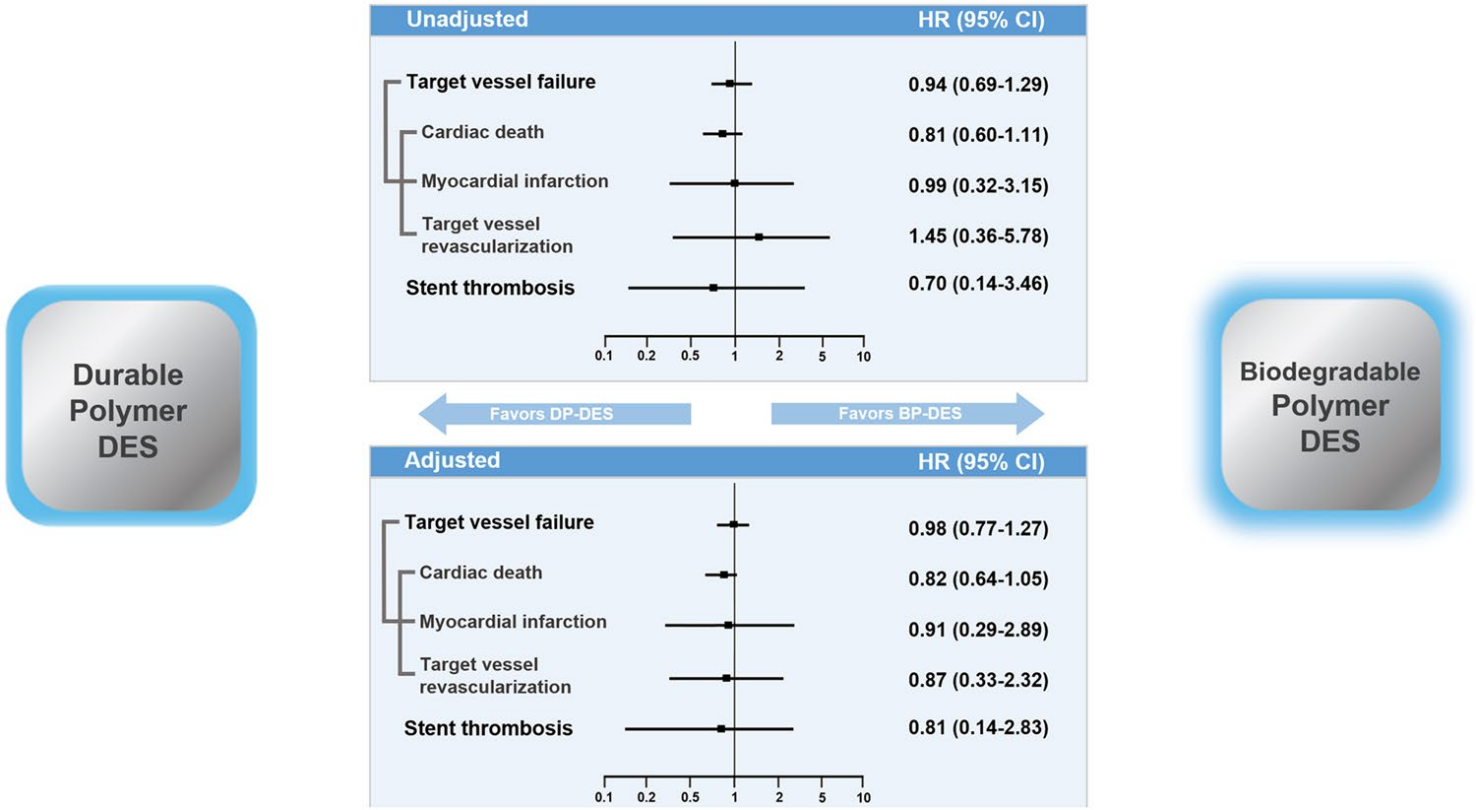
DP-DES vs. BP-DES in AMI Complicated by Cardiogenic Shock: Summary of 12-month Outcomes. *BP-DES* bioabsorbable polymer drug-eluting stent, *CI* confidence interval; *DP-DES* durable polymer drug-eluting stent, *HR* hazard ratio.

After IPTW adjustment, the incidence of TVF was similar in the two groups (140 [28.1%] vs. 123 [25.1%], HR 0.98, 95% CI 0.77–1.27; *p* = 0.899). The incidence of all-cause death (*p* = 0.296), cardiac death (*p* = 0.121), myocardial infarction (*p* = 0.873), target vessel revascularization (*p* = 0.782), stent thrombosis (*p* = 0.441), any revascularization (*p* = 0.061), and re-hospitalization due to heart failure (*p* = 0.457) was not significantly different between the two groups (Table 3 and Fig. 3) (see Supplementary Fig. S1 online).

### Subgroup analysis

**Figure 4:**
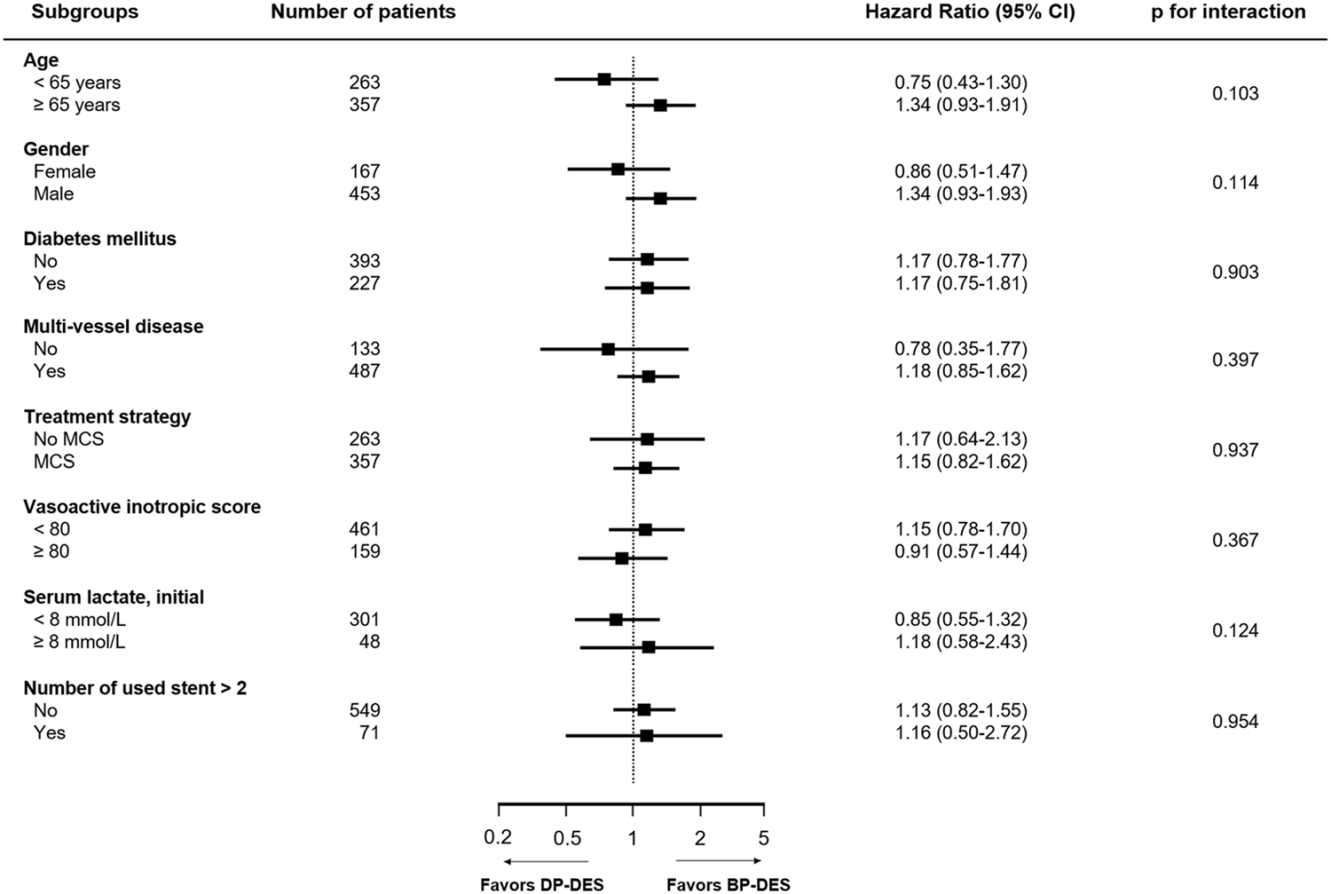
Comparative unadjusted hazard ratios of TVF between DP-DES and BP-DES. TVF was defined as a composite of cardiac death, myocardial infarction, and target vessel revascularization. *BP-DES* bioabsorbable polymer drug-eluting stent, *CI* confidence interval, *DP-DES* durable polymer drug-eluting stent, *MCS* mechanical circulatory support, *TVF* target vessel failure.

We performed subgroup analyses to evaluate the association between polymer technology in DESs and TVF in various situations. The prognostic effect of polymer technology was consistent across subgroups regardless of age, sex, vasoactive inotropic score, need for MCS, serum lactate level, multi-vessel disease, diabetes mellitus, or number of DESs used (Fig. 4).

## Discussion

This study investigated the clinical outcomes of DP-DESs compared to BP-DESs in AMI patients with CS utilizing data from a recent, large-scale, dedicated, real-world multicenter CS registry. The key finding of the present study was that there were no significant differences in risk of TVF consisting of cardiac death, myocardial infarction, or target vessel revascularization at 12 months between the DP-DES and the BP-DES groups, which was consistent across subgroups by use of MCS as well as various clinical factors. To the best of our knowledge, this study represents a novel investigation into the efficacy and safety of newer-generation DP-DESs compared to newer-generation BP-DESs in AMI patients with CS. In recent years, DESs have undergone refinements that include using biodegradable polymers to deliver anti-proliferative substances and reducing strut thickness of the metallic stent platform. These design improvements of the stent help to alleviate arterial injury, inflammation, and thrombogenicity; aid in the re-endothelialization process; and decrease neointimal hyperplasia^14^. Therefore, patients receiving newer-generation DESs exhibit better long-term clinical outcomes compared to those receiving previous DESs^15,16^. Especially, findings supporting the non-inferiority of biodegradable-polymer sirolimus-eluting stents compared to second-generation DESs^17–19^ have recently been challenged by accumulating evidence suggesting superiority over durable-polymer everolimus-eluting stents in terms of device-oriented clinical outcomes in patients with ACS^6,20,21^.

However, comparison of DP-DESs and BP-DESs has shown controversial results in patients receiving PCI. Iglesias et al. compared BP-DESs and DP-DESs in patients with ACS and showed that the BP-DES was superior to the DP-DES in terms of target lesion failure, emphasizing the benefits of complete polymer degradation, which reduces thrombogenicity and facilitates endothelialization. The frequency of target lesion revascularization, which was the primary factor causing the difference, was much higher in the DP-DES group during the first three months but was similar in the two types of polymer DESs over the 12-month follow-up period^20^. In contrast, Kim et al. demonstrated that the risk of patient-oriented clinical outcome was similar between the DP-DES and BP-DES groups, whereas the risk of a composite of cardiac death, target-vessel myocardial infarction, or target lesion revascularization was lower in the DP-DES group, driven primarily by the lower incidence of target lesion revascularization. The higher risk of target lesion revascularization in the BP-DES group was attributed to the characteristics of BP-DESs becoming similar to bare-metal stent after polymer degradation, thereby increasing the risk of late restenosis^6^. In a recent real-world study involving a database of more than 95,000 stents, there was no additional clinical benefit associated with BP-DES over DP-DES during a two-year follow-up period, suggesting that the lack of incremental benefit with BP-DESs was due to the enhanced biocompatibility of polymers, as well as reduced strut thickness in DP-DESs^5^. These previous studies included a significant proportion of patients with stable ischemic heart disease without CS. Given the high acuity and critical condition of CS, patients with AMI complicated by CS exhibit much higher mortality than those with stable ischemic heart disease and experience most clinical adverse events early in the clinical course^22^, making the clinical benefits of improved polymer technology less prominent. Therefore, the effects of these differences in polymer technology on clinical outcomes in this population have remained unclear, as there is insufficient data on the efficacy and safety of DP-DESs compared to BP-DESs for AMI complicated by CS.

This study focused on the specific concerns surrounding the use of newer-generation DESs and revealed that DP-DESs were not inferior to BP-DESs in terms of TVF and all individual components of TVF 12 months after index PCI. Even though the study population was comprised of patients with AMI complicated by CS who had increased risk of thrombotic formation and higher incidence of myocardial infarction, stent thrombosis, and target vessel revascularization compared to patients with stable ischemic heart disease receiving PCI, both polymer types showed better outcomes than expected. Our study determined that, while the various polymer technologies utilized in newer-generation DESs offer distinct procedural advantages, they do not lead to different clinical outcomes over 12 months. Furthermore, in subgroup analysis, the similarity of TVF between the two polymer DES groups was consistent across various clinical factors, corresponding well with findings of earlier studies that established no significant interactions of clinical outcomes between types of polymer technology according to subgroup^6,19^. In particular, to assess the potential interactions between types of polymer technology and the severity of CS, our study focused on clinical variables such as vasoactive inotropic score and MCS requirement due to the previously established link between high vasoactive inotropic score or need for MCS and unfavorable clinical outcomes in patients with CS^9,23^. In our study, there were no significant interactions between polymer technology and clinical outcomes according to CS severity.

The present study has some limitations. First, it had a non-randomized design and relied on observational data from multiple centers, which may have introduced selection bias and unmeasured confounding factors that could impact the results. Although optimal pharmacological therapy was recommended, our study lacked detailed information on post-PCI medical treatments during the follow-up period, including duration of dual antiplatelet therapy. Moreover, the choice of revascularization strategy or application of MCS was left to the operator’s discretion, possibly introducing selection bias. Due to the retrospective nature of our registry, we could not thoroughly elucidate the mechanism underlying the association of clinical outcomes with different DES platforms, which may have limited our results. Second, the limited sample size could be a contributing factor to the absence of significant interactions observed in certain subgroup analyses. As a result, the findings from this study should be considered as hypothesis-generating and warrant confirmation through well-designed randomized trials. Third, the rate of nonfatal events was comparatively lower than the rate of death. To ensure accurate data entry into the electronic case report form, periodic site monitoring, active follow-up, and source document auditing in individual centers were conducted, but the possibility of missed events cannot be completely ruled out. Fourth, we were not able to evaluate the roles of the design of the stent, including its architecture and strut thickness, or the type of anti-proliferative drug and its release kinetics, which might have affected clinical results. The DP-DES group consisted exclusively of patients receiving second-generation DESs, whereas the BP-DES group included those with early-generation stents such as the Biomatrix and the Nobori stents. The heterogeneous stent configuration remains a significant limitation. Finally, the analysis confined to a 12-month follow-up is a limitation of this study. The impact of polymer technology on stent performance may not be evident within this 12-month period due to the pathophysiology of neoatherosclerosis and the prolonged degradation time of biodegradable polymer, considering previous studies reporting the long-term superiority of BP-DESs over DP-DESs for AMI patients^24,25^. To validate the efficacy and safety of DP-DESs and BP-DESs, a longer follow-up duration is needed.

## Conclusions

We found no significant difference in clinical outcomes between the DP-DES and BP-DES groups in AMI patients complicated by CS from a large-scale, dedicated CS registry. The clinical outcomes did not seem to be affected by polymer technology for AMI with CS, which is representative of a high thrombus burden with a risk of undersized stenting. In the current DES era, focusing on the optimization of CS therapy itself could improve clinical outcomes.

### Supplementary Information

Supplementary Figure S1.

Supplementary Legends.

## Data Availability

The datasets generated during or analysed during the current study are available from the corresponding author on reasonable request.
