# Supplementary figures and images for: Efficacy and safety of durable versus biodegradable polymer drug-eluting stents in patients with acute myocardial infarction complicated by cardiogenic shock
Source: Sci Rep. 2024 Mar 15;14:6301. doi: 10.1038/s41598-024-56925-2 (PMC10943207; doi:10.1038/s41598-024-56925-2)

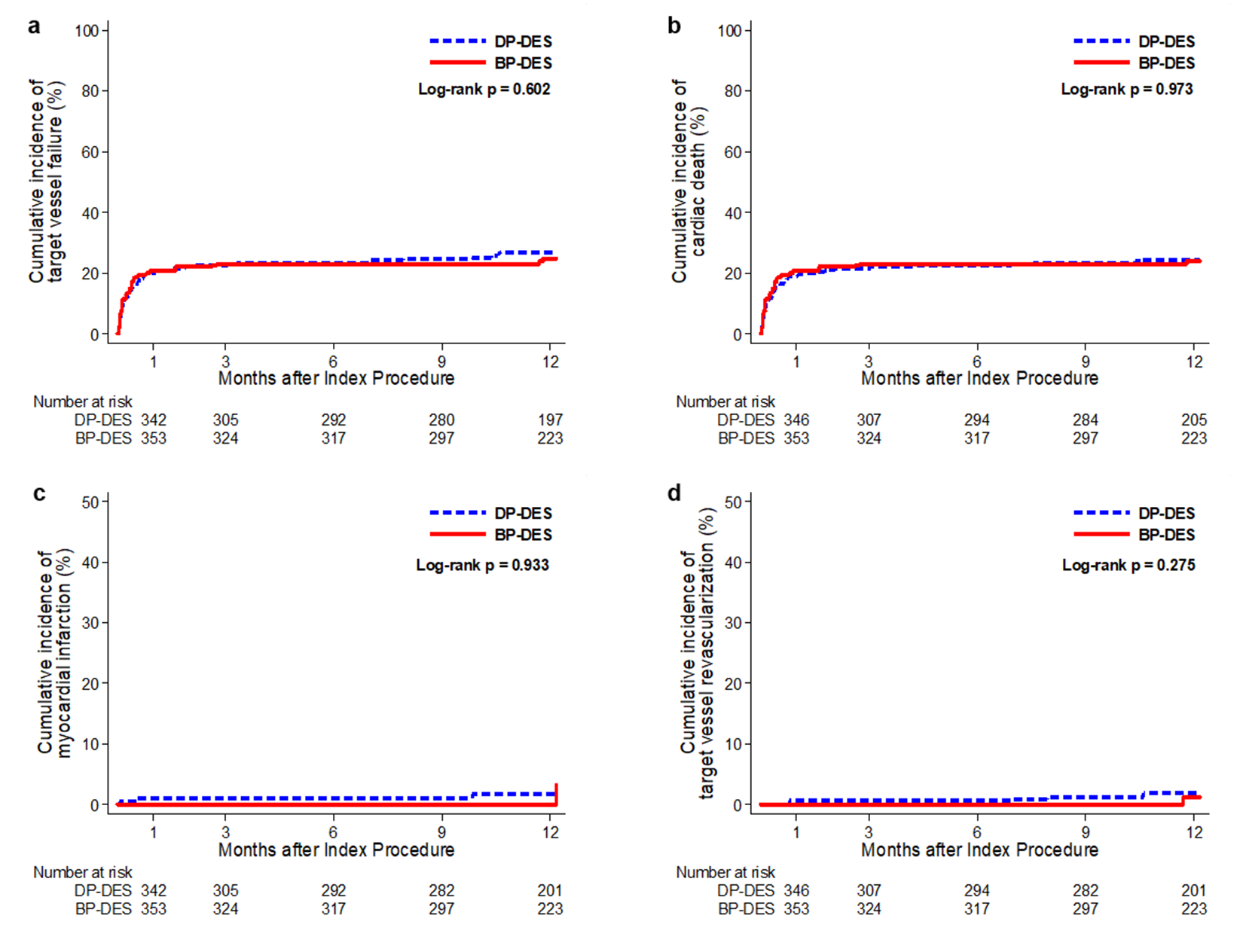

Supplement: Supplementary file 1 — Supplementary Figure S1. [file 41598_2024_56925_MOESM1_ESM.tif]
