# Supplementary material for: Efficacy and safety of durable versus biodegradable polymer drug-eluting stents in patients with acute myocardial infarction complicated by cardiogenic shock
Source: Sci Rep. 2024 Mar 15;14:6301. doi: 10.1038/s41598-024-56925-2 (PMC10943207; doi:10.1038/s41598-024-56925-2)
Supplement: Supplementary file 2 — Supplementary Legends. [file 41598_2024_56925_MOESM2_ESM.pdf]

Supplementary Information

Efficacy and Safety of Durable Versus Biodegradable Polymer Drug-Eluting Stents in Patients with Acute Myocardial Infarction Complicated by Cardiogenic Shock

Running title: Durable- vs. biodegradable polymer DES in CS

Woo Jin Jang, MD[1]; Ik Hyun Park, MD[2]; Ju Hyeon Oh, MD[2]; Ki Hong Choi, MD[3]; Young Bin Song, MD[3]; Joo-Yong Hahn, MD[3]; Seung-Hyuk Choi, MD[3]; Hyeon-Cheol Gwon, MD[3]; Chul-Min Ahn, MD[4]; Cheol Woong Yu, MD[5]; Hyun-Joong Kim, MD[6]; Jang-Whan Bae, MD[7]; Sung Uk Kwon, MD[8]; Hyun Jong Lee, MD[9]; Wang Soo Lee, MD[1]; Jin-Ok Jeong, MD[10]; Sang-Don Park, MD[11]; Jeong Hoon Yang, MD[3*]

[1]Division of Cardiology, Chung-Ang University Hospital, Seoul, Republic of Korea

[2]Department of Cardiology, Samsung Changwon Hospital, Sungkyunkwan University School of Medicine, Changwon, Republic of Korea

[3]Division of Cardiology, Heart Vascular Stroke Institute, Samsung Medical Center, Sungkyunkwan University School of Medicine, Seoul, Republic of Korea

[4]Division of Cardiology, Severance Cardiovascular Hospital, Yonsei University College of Medicine, Seoul, Republic of Korea

[5]Division of Cardiology, Department of Internal Medicine, Korea University Anam Hospital, Seoul, Republic of Korea.

[6]Division of Cardiology, Konkuk University Medical Center, Seoul, Republic of Korea

[7]Division of Cardiology, Chungbuk National University College of Medicine, Cheongju, Republic of Korea

[8]Division of Cardiology, Ilsan Paik Hospital, University of Inje College of Medicine, Seoul, Republic of Korea

[9]Division of Cardiology, Sejong General Hospital, Bucheon, Republic of Korea

[10]Division of Cardiology, Chungnam National University Hospital, Daejeon, Republic of Korea

[11]Division of Cardiology, Inha University Hospital, Incheon, Republic of Korea

Dr. Jang and Dr. Park equally contributed to this work as first authors.

Correspondence:

* Jeong Hoon Yang, MD, PhD

Division of Cardiology, Department of Critical Care Medicine and Medicine,

Samsung Medical Center, Sungkyunkwan University School of Medicine,

81 Irwon-ro, Gangnam-gu, Seoul, 135-710, Republic of Korea

Phone: +82-2-3410-1768, FAX: +82-2-2148-7088

Email: jhysmc@gmail.com

(1) Supplementary Figure S1 legend

Supplementary Figure S1. Time-to-event Kaplan-Meier survival curves of primary outcome according to polymer after inverse probability of treatment weighting adjustment

(a) Kaplan-Meier curves for target vessel failure. (b) Kaplan-Meier curves for cardiac death. (c) Kaplan-Meier curves for myocardial infarction. (d) Kaplan-Meier curves for target vessel revascularization.

TVF was defined as a composite of cardiac death, MI, and target vessel revascularization.

BP-DES = bioabsorbable polymer drug-eluting stent; DP-DES = durable polymer drug-eluting stent.
